# Preparation of Chicken Anemia Virus (CAV) Virus-Like Particles and Chicken Interleukin-12 for Vaccine Development Using a Baculovirus Expression System

**DOI:** 10.3390/pathogens8040262

**Published:** 2019-11-23

**Authors:** Ta-Yuan Tseng, Yee-Chen Liu, Yu-Chen Hsu, Poa-Chun Chang, Ming-Kun Hsieh, Jui-Hung Shien, Shan-Chia Ou

**Affiliations:** 1 Graduate Institute of Microbiology and Public Health, College of Veterinary Medicine, National Chung Hsing University, Taichung 402, Taiwan; darrenmike@gmail.com (T.-Y.T.); ame107223510@gmail.com (Y.-C.L.); tsula17@gmail.com (Y.-C.H.); pcchang@mail.nchu.edu.tw (P.-C.C.); mhsieh@dragon.nchu.edu.tw (M.-K.H.); 2 Animal Disease Diagnostic Center, College of Veterinary Medicine, National Chung Hsing University, Taichung 402, Taiwan; 3 Department of Veterinary Medicine, College of Veterinary Medicine, National Chung Hsing University, Taichung 402, Taiwan

**Keywords:** chicken infectious anemia vaccine, chicken anemia virus, chicken IL-12, adjuvant, virus-like particle, vaccine development

## Abstract

Chicken infectious anemia (CIA) is a poultry disease that causes huge economic losses in the poultry industry worldwide. Commercially available CIA vaccines are derived from wild-type chicken anemia viruses (CAVs) by serial passage in cells or chicken embryos. However, these vaccinal viruses are not completely attenuated; therefore, they can be transmitted vertically and horizontally, and may induce clinical symptoms in young birds. In this study, we sought to eliminate these issues by developing a subunit vaccine exploiting the CAV structural proteins, engineering recombinant baculovirus-infected *Spodoptera frugiperda* (*Sf*9) cells that contained both the viral protein 1 (VP1) and VP2 of CAV. Moreover, we produced single-chain chicken interleukin-12 (chIL-12) in the same system, to serve as an adjuvant. The recombinant VP1 was recognized by chicken anti-CAV polyclonal antibodies in Western blotting and immunofluorescence assays, and the bioactivity of the recombinant chIL-12 was confirmed by stimulating interferon-γ (IFN-γ) secretion in chicken splenocytes. Furthermore, the ability of the recombinant VP1 to generate self-assembling virus-like particles (VLPs) was confirmed by transmission electron microscopy. Specific pathogen-free (SPF) chickens inoculated with VLPs and co-administered the recombinant chIL-12 induced high CAV-specific antibodies and cell-mediated immunity. Taken together, the VLPs produced by the baculovirus expression system have the potential to be a safe and effective CIA vaccine. Finally, we demonstrated the utility of recombinant chIL-12 as an adjuvant for poultry vaccine development.

## 1. Introduction

Chicken infectious anemia (CIA) causes severe economic losses in the poultry industry worldwide. The causative pathogen, chicken anemia virus (CAV), induces clinical anemia in young chicks when they lack protective maternal antibodies [1]. However, infected chickens older than three weeks show subclinical immunosuppression, which results in susceptibility to other infections and poor vaccine responses [2,3].

CAV, a non-enveloped DNA virus, is a member of the *Gyrovirus* genus and the family of *Anelloviridae* [4]. The genome of CAV is a circular, single-stranded, negative-sense DNA approximately 2.3 kb in length [5,6,7]. CAV consists of three partially overlapping open reading frames (ORFs) that encode three viral proteins (VPs). VP1, the major structural capsid protein, plays an important role in antigenicity, and induces neutralization antibodies in hosts [8]. The non-structural VP2 acts as a scaffold protein to assist the correct assemblage of VP1 with a protein phosphatase [9,10]. The co-expression of VP1 and VP2 in the same cells is necessary for recombinant VP1 to form correctly and induce neutralizing antibodies in inoculated chickens [9,11]. VP3, also known as apoptin, induces apoptosis in chicken thymocytes and lymphoblastoid T cells [12].

Currently, commercially available CIA vaccines are derived from wild strains that have been serially passaged in cells or chicken embryos for intensive attenuation [13]. However, these vaccines are not completely attenuated; therefore, they may be transmitted vertically or horizontally, causing clinical signs in young chicks. Moreover, these CIA vaccines can revert to virulent phenotypes after chicken-to-chicken transmission in the field [14,15]. As a result, CIA vaccine administration is recommended to breeders for chicks older than six weeks of age, and again four weeks prior to laying, so as to confer maternally-derived antibody protection to offspring [1].

To circumvent issues of incomplete attenuation, several studies have attempted to develop subunit vaccines. A recent study reported that the codon-optimized VP1 of CAV increases the production of recombinant VP1 in *E. coli* [16]. The VP1 of CAV can also be expressed in a plant system, which may yield CAV vaccines after optimizing the VP1 expression level [17]. Neutralizing antibodies are induced against CAV when chickens are inoculated with plasmid-based DNA vaccines containing *VP1* and *VP2* genes in a vector simultaneously [18,19]. Previous studies revealed that triplicate-inactivated CAVs administered to hens at titers of 10^7.5^TCID_50_ or 7.9 × 10^17^ copies/μL induce sufficient maternal antibodies to protect offspring from CAV symptoms after experimental challenges; however, the difficulty of achieving a sufficiently high virus titer for an effective inactivated vaccine remains a challenge to vaccine development [20,21].

In addition to vaccine optimization through recombinant systems, adjuvants, such as cytokines, can enhance vaccine efficacy by increasing the immunogenicity of antigens, stimulating host humeral or cellular immune responses [22,23]. Interleukin-12 (IL-12) establishes type-1 T helper cell (Th1) immune responses crucial to the elimination of intracellular pathogens [24]. Chicken IL-12 has been cloned, and its structure and function are similar to that of mammalian IL-12 [25]. When expressed in plant or chicken DF-1 cells, the single chain of chicken IL-12p70, which contains p40 and p35 subunits, stimulates the secretion of interferon-γ (IFN-γ) and the proliferation of chicken splenic lymphocytes [26,27]. In addition, N-linked glycosylation is critical for the bioactivity of mammalian and chicken IL-12 [26,28]; because bacteria are rarely capable of this process, eukaryotic expression systems are likely ideal for the production of recombinant chicken IL-12. 

To ameliorate the disadvantages of the existing live attenuated CIA vaccines, in this study, the VP1 and VP2 of CAV, along with chicken IL-12, were expressed using a baculovirus-insect cell expression system. Western blotting and indirect immunofluorescence assays (IFA) verified the expression of these recombinant proteins in *Spodoptera frugiperda* (*Sf*9) cells. Recombinant IL-12 functioned appropriately as a biological adjuvant, inducing high levels of IFN-γ in chicken splenocytes. Moreover, recombinant VP1 combined with chicken IL-12 induced high specific CAV antibody titers in vaccinated chickens, suggesting that recombinant VP1 co-administrated with chicken IL-12 is a promising vaccine candidate for CAV in chickens.

## 2. Results

### 2.1. Expression of Recombinant Proteins

**Figure 1:**
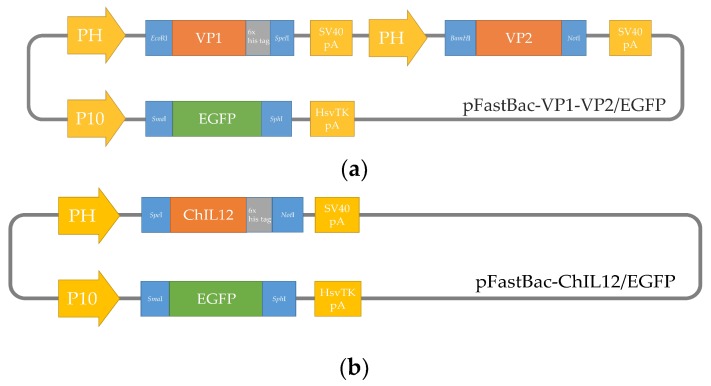
Construction of recombinant plasmids, with codon-optimized viral protein 1 (VP1) and VP2 of chicken anemia virus (CAV) and chicken interleukin-12 (IL-12) genes inserted into pFastBac^TM^ Dual plasmids. (**a**) pFastBac–VP1–VP2/EGFP plasmid: VP1 and VP2 of CAV were inserted into independently separated expression cassettes driven by polyhedron (PH) promoters, and EGFP was cloned into the P10 promoter-expressing cassette. (**b**) pFastBac-ChIL-12/EGFP plasmid: chicken IL-12 was inserted into the PH promoter-expressing cassette.

**Figure 2:**
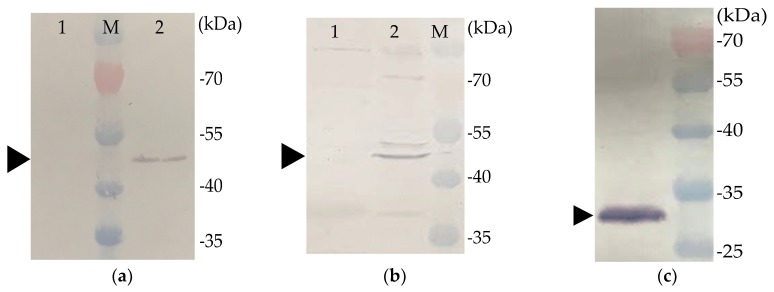
Western blotting analysis of rVP1 and rVP2. Proteins from baculovirus B-VP1-/B-VP2-infected *Spodoptera frugiperda* (*Sf*9) cell lysates were detected by an (**a**) anti-His-tagged monoclonal antibody, or (**b**) chicken anti-CAV polyclonal antibodies as primary antibodies. The rVP1 was recognized by the antibodies and showed a ~50 kDa band (arrow). Lane 1, control *Sf*9 cell lysate; lane 2, B-VP1-/B-VP2-infected *Sf*9 cell lysate. (**c**) The rVP2 was recognized by anti-*E. coli*-expressed VP2 polyclonal antibodies as primary antibodies, and showed a ~30 KDa band (arrow).

**Figure 3:**
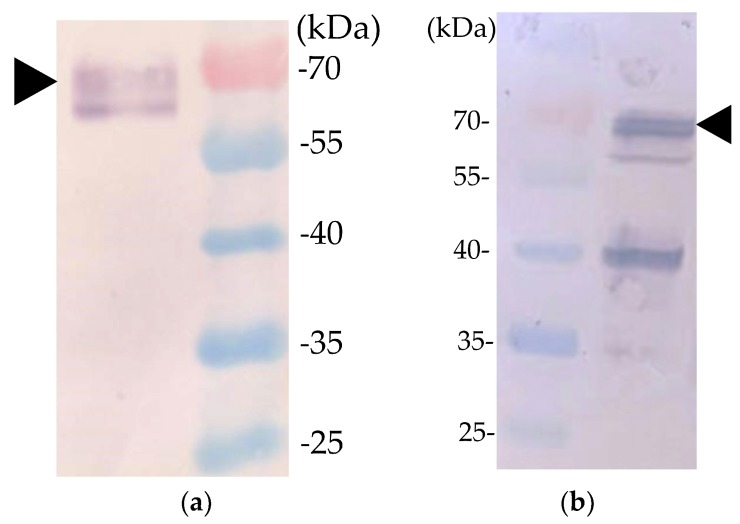
Western blotting analysis of rchIL-12. Proteins from baculovirus (B-chIL-12)-infected *Sf*9 cell lysates were detected by an (**a**) anti-His-tagged monoclonal antibody, or (**b**) anti-*E. coli*-expressed chIL-12 as the primary antibodies. The recombinant protein was recognized by the antibodies and showed a ~70 kDa band (arrow).

**Figure 4:**
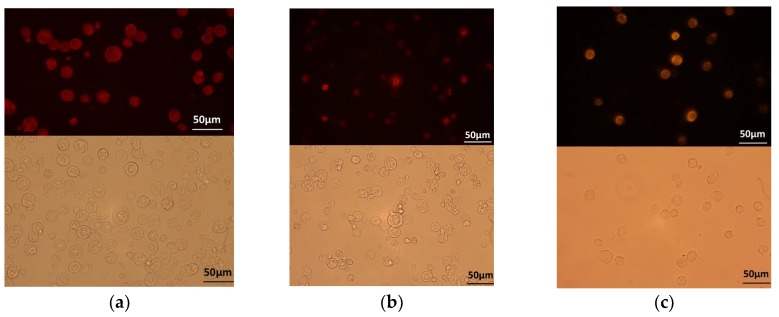
Immunofluorescence assays (IFA) of recombinant proteins. Baculovirus-infected *Sf9* cells expressing recombinant VP1 were detected with (**a**) chicken anti-CAV polyclonal primary antibodies and goat anti-chicken IgG secondary antibodies (red). (**b**) anti-His-tagged monoclonal primary antibody and goat anti-mouse IgG secondary antibodies (red). (**c**) Baculovirus-infected *Sf*9 cells expressing recombinant IL-12 were detected with mouse anti-*E. coli*-expressed IL-12 polyclonal primary antibodies and goat anti-mouse IgG secondary antibodies (yellow).

Previous studies revealed that the VP1 and VP2 of CAV must be co-expressed or co-synthesized by *Sf*9 cells to form neutralizing antibody-recognized epitopes in VP1 of CAV [9,11]. We therefore constructed a recombinant baculovirus that contained two expression cassettes for expressing VP1 and VP2 simultaneously (Figure 1). rVP1, rVP2, and rchIL-12 were detected with Western blotting or IFA. An approximately 50-KDa protein was detected in the cell lysates of B-VP1-/B-VP2-infected *Sf*9 cells using Western blot with an anti-His-tagged monoclonal antibody or chicken anti-CAV polyclonal antibodies as probes. Moreover, the expression of VP2 in *Sf*9 cells was confirmed by the anti-*E. coli*-expressed VP2 polyclonal antibodies as primary antibody, and showed an approximately 30-KDa protein using Western blot (Figure 2). The results revealed that VP1 and VP2 were expressed in B-VP1-/B-VP2-infected *Sf*9 cells simultaneously. In addition, an approximately 70-KDa protein was detected in the cell lysate of B-chIL-12-infected *Sf*9 cells by Western blot, with an anti-His-tagged monoclonal antibody or polyclonal antibodies recognizing *E. coli*-expressed chIL-12. Moreover, a degradation product of rchIL-12 below 70 KDa was also detected by the anti-His-tagged monoclonal antibody (Figure 3). The expression of rVP1 in *Sf*9 cells was confirmed by IFA with chicken anti-CAV polyclonal antibodies and an anti-His-tagged monoclonal antibody. Moreover, rchIL-12 was recognized by the anti-*E. coli*-expressed chIL-12 antibodies (Figure 4). These results confirmed that the CAV rVP1 and rchIL-12 were successfully expressed in *Sf*9 cells.

### 2.2. Virus-Like Particles (VLPs) Formed by Recombinant VP1 Protein

**Figure 5:**
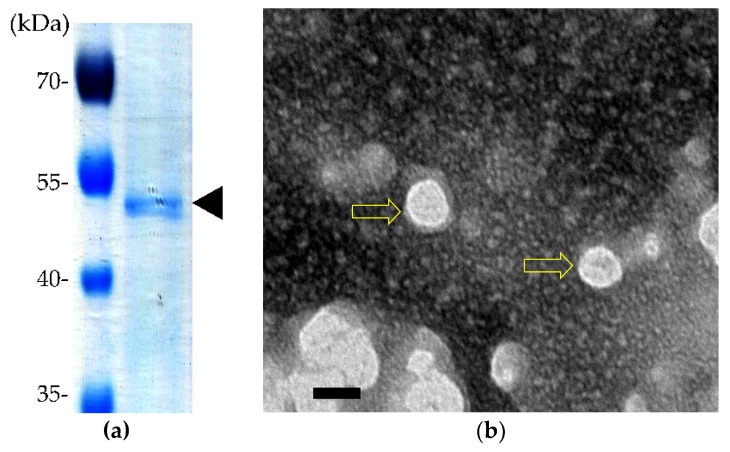
The SDS-PAGE of purified rVP1 and a TEM analysis of the CAV virus-like particle (VLP) formation. (**a**) rVP1 was collected from a nickel affinity (Ni-NTA resin) column, and the purified rVP1 was separated by SDS-PAGE. (**b**) The purified, negative-stained rVP1 self-assembled into particles approximately 25 nm in diameter, a size consistent with CAV particles (yellow hollow arrow). Bar = 20 nm.

rVP1 purified from B-VP1/VP2-infected *Sf*9 cells was verified by SDS-PAGE (Figure 5a). Moreover, after negative staining, VLPs were observed with transmission electron microscopy (TEM), which indicated that the recombinant VP1 self-assembled into particles approximately 25 nm in diameter, a size consistent with the CAV particles (Figure 5b). 

### 2.3. Bioactivity of rchIL-12

**Figure 6:**
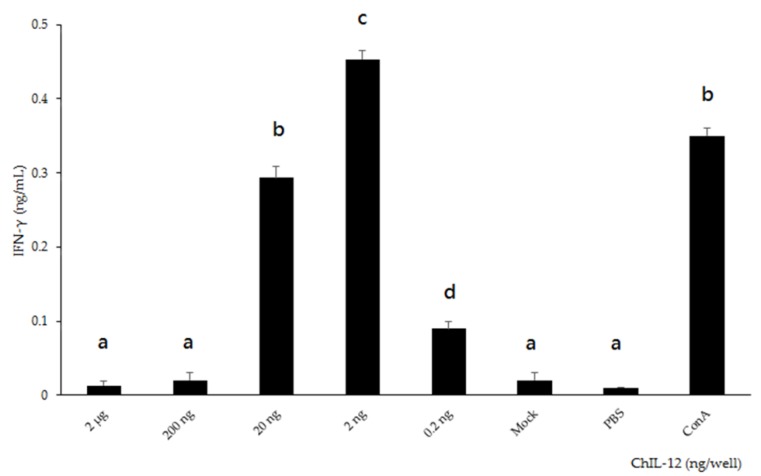
Bioactivity assay of rchIL-12. Chicken splenocytes were treated with serially diluted, purified rchIL-12 (2 μg–0.2 ng/well), *Sf*9 cell lysate only (mock), ConA, or phosphate-buffered saline (PBS), and interferon-γ (IFN-γ) concentration in the supernatant was quantified. Data are displayed as the mean ± standard deviation (SD). The different letters (a, b, c, and d) above the bars indicate a statistically significant difference between the different groups (*p* < 0.05).

The splenocytes from specific pathogen-free (SPF) chickens were treated, in triplicate, with serially diluted (2 μg–0.2 ng), purified rchIL-12, and the IFN-γ in the supernatant was quantified. The splenocytes treated with rchIL-12 secreted IFN-γ, and the IFN-γ induction significantly increased concomitantly with the rchIL-12 concentration, from an average of 0.09 ng/mL at 0.2 ng of rchIL-12, and reaching a peak averaging 0.45 ng/mL at 2 ng of rchIL-12. The splenocytes treated with *Sf*9 cell lysate only (mock) and phosphate-buffered saline (PBS) showed very low IFN-γ levels (Figure 6). These results suggest that insect cells produced bioactive chIL-12, because *Sf*9-expressed rchIL-12 stimulated the production of IFN-γ in chicken splenocytes. 

### 2.4. Immune Response of VLP-Vaccinated Chickens

**Figure 7:**
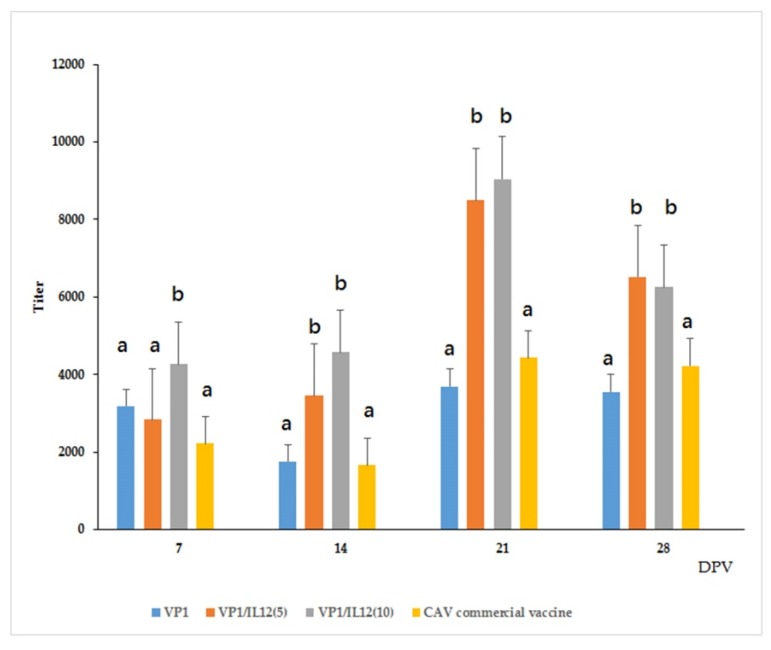
CAV antibody titers detected from rVP1-vaccinated chickens using a commercial ELISA kit. SPF chickens were vaccinated with VP1, VP1/chIL-12(5), VP1/chIL-12(10), or commercial vaccine, and serum samples were collected at 7, 14, 21, and 28 days post-primary vaccination (dpv). Serum samples pre-vaccination and post-administration in the PBS group showed no detectable CAV antibody. Data are displayed as geometric mean ± SD. Different letters (a and b) above the bars indicate a statistically significant difference between the different groups (*p* < 0.05).

The SPF chickens were immunized according to one of the following five treatments: (1) rVP1 only, (2) rVP1 co-administered with 5 ng rchIL-12 [VP1/IL-12(5)], (3) rVP1 co-administered with 10 ng rchIL-12 [VP1/IL-12(10)], (4) a commercial vaccine (positive control), or (5) PBS only (negative control). The CAV-specific antibodies were quantified pre- and post-vaccination; serum samples before immunization in all groups and for the PBS-only group showed no detectable CAV antibodies. Seven days post-primary vaccination (dpv), antibodies were detected in the serum samples from every immunized group, and the mean titer from the VP1/IL-12(10) group was significantly higher than that of theother groups (*p* < 0.05). At 14 dpv, the CAV antibody titers for the VP1/IL-12(5)- and VP1/IL-12(10)-treated groups were not significantly different (average of 3459 and 4570, respectively), and they were significantly higher than those of rVP1-only- and commercial vaccine-treated groups (*p* < 0.05). At 21 dpv, the mean antibody titers were not significantly different between VP1/IL-12(10) and VP1/chIL-12(5) (average of 9034 and 8497, respectively), but both were significantly higher than those of the rVP1-only or commercial vaccine groups (*p* < 0.05). At 28 dpv, the titers declined slightly in each group, but the rVP1 plus rchIL-12 groups had significantly higher antibody titers than the rVP1-alone or the commercial vaccine groups (*p* < 0.05). These results indicated that rVP1 induced CAV-specific antibodies in immunized chickens, and that the rchIL-12 used here enhanced immune responses in vaccinated chickens and served as a vaccine adjuvant. Furthermore, the chickens in the VP1/chIL-12(5) group produced similar CAV antibody titers to that of the VP1/chIL-12(10) group at 14, 21, and 28 dpv (Figure 7), suggesting that a 5-ng dose of rchIL-12 is sufficient to stimulate anti-CAV antibodies in this CAV vaccine. 

### 2.5. Antigen-Specific Splenocyte Proliferation and IFN-γ Secretion Assays

**Figure 8:**
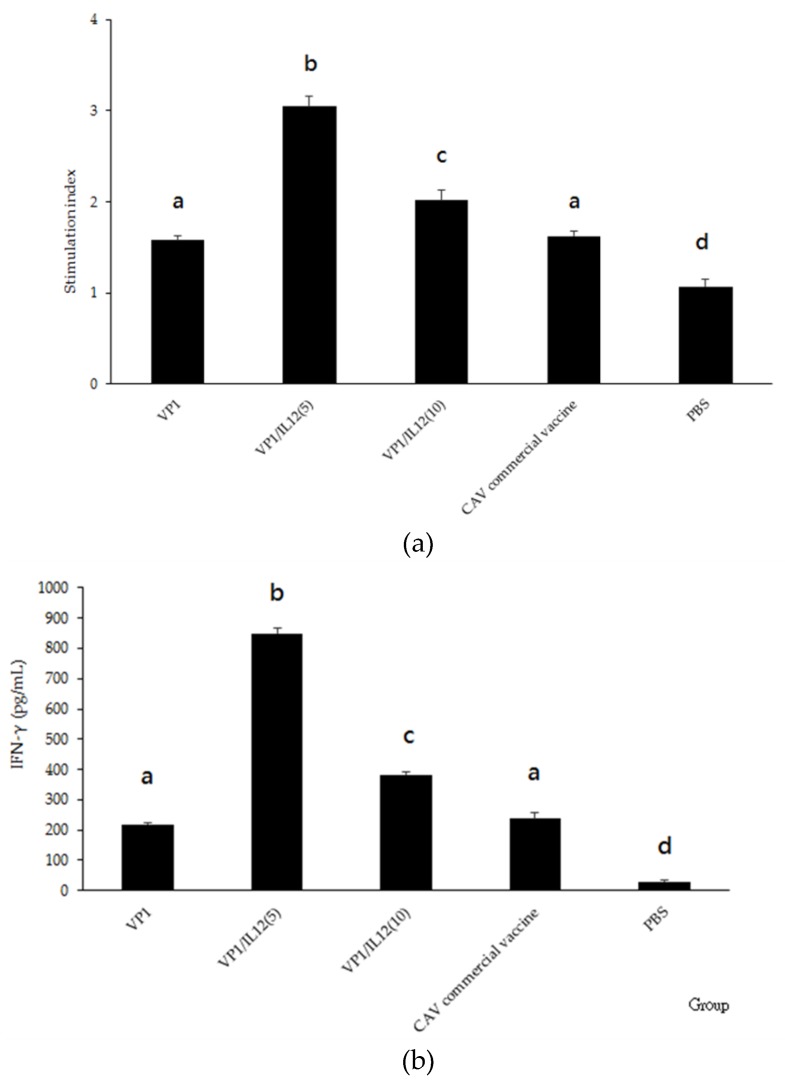
Antigen-specific splenocyte proliferation and IFN-γ secretion assays. At 28 dpv, chicken spleens were collected from each experimental group, and the splenocytes were treated with purified rVP1. (**a**) Splenocyte proliferation assay with the stimulation index (SI) values. (**b**) Concentration of IFN-γ. Data are displayed as mean ± SD. Different letters (a, b, c, and d) above the bars indicate a statistically significant difference between the different groups (*p* < 0.05).

The splenocytes collected from the experimental groups were treated with purified rVP1, and the proliferation response at 28 dpv was quantified. Vaccine-immunized groups showed significantly higher stimulation index (SI) values than the PBS group (*p* < 0.05; Figure 8a). The splenocytes collected from the VP1/chIL-12(5) group produced the highest SI value (*p* < 0.05). Moreover, the IFN-γ production by the splenocytes from the rVP1-treated groups was also higher than that of PBS-treated groups, with the VP1/chIL-12(5) group producing the highest concentration of IFN-γ (*p* < 0.05; Figure 8b). The spleen cells from the vaccinated chickens showed proliferation and secreted IFN-γ in vitro, indicating that the rVP1 subunit vaccine stimulated the cell-mediated immunity in these chickens. 

## 3. Discussion

Currently, commercially available live attenuated CIA vaccines have several shortcomings [1]. As a result, a safe, high quality, and economical CIA vaccine is needed in order to improve chicken production. In this study, CAV structural proteins and chicken IL-12 were expressed in the *Sf*9 insect cell system. Recombinant VP1 induced CAV-specific antibodies in immunized chickens. In addition, the bioactivity assays showed that the chicken IL-12 expressed by this system served as a vaccine adjuvant. Finally, this *Sf*9-expressed recombinant VP1 formed the self-assembled VLPs, as observed by TEM, necessary to generate neutralizing epitopes (Figure 5). This is the first report to confirm by TEM that the VLPs of CAV can be generated using in vitro protein expression systems. 

Prokaryotic expression systems are an attractive means to produce CAV VP1, and several studies have attempted *E. coli* expression systems. Because the N-terminus of VP1 is cytotoxic to *E. coli* [29], N-terminal-deleted VP1 increases yields in *E. coli* [30], and the full VP1 protein can be generated in *E. coli* by optimizing codons and fusing the GST protein in VP1 [16]. VP1 expressed by this strategy can be recognized by CAV-positive chicken serum; however, the immunogenicity of this *E. coli* expressed recombinant VP1 needs further investigation. 

In addition to the attenuated virus vaccines, the existing CAV vaccine development strategies include DNA and inactivated virus vaccines. CAV DNA vaccines have been described previously, including *VP1* and *VP2* cloned into a mammalian expression vector, *VP1* fused with the *VP22* gene of Marek’s disease virus, and a CAV DNA vaccine co-administered with *E. coli*-expressed truncated high mobility group box 1 (HMGB1) protein [18,19,31], all of which are capable of producing detectable antibody titers. However, DNA vaccines are time- and labor-intensive in chickens, which must be vaccinated multiple times in order to reach a sufficient antibody titer. Alternatively, inactivated CAV vaccines derived from wild types of CAV induced high serum titers in vaccinated hens, and protected their progeny against challenges [20,21]; however, CAV grows slowly by in vitro propagation in MDCC-MSB1 cells or chicken embryos [13], and is therefore a labor-intensive and time-consuming means of vaccine preparation. Thus, a more economical and reliable vaccination system is needed by global poultry industries.

In this study, CAV VLPs induced similar antibody titers to commercial vaccines. In addition, when VLPs were co-administrated with rchIL-12, significantly higher antibody titers were induced, relative to the VP1-only and commercial vaccine groups (Figure 7). Previous studies suggest that CAV antibody titers above 5000 in hens protect progeny against CAV infection for the first four weeks after hatching [32,33]. In our study, the chickens receiving VP1/chIL-12(5) and VP1/chIL-12(10) vaccines induced titers in excess of 5000, indicating that CAV VLP combined with rchIL-12 is a high-efficacy vaccine candidate for CAV. 

In this study, we described how IL-12, an immune regulator that stimulates Th1 immune responses [24], functioned as an adjuvant for our recombinant CAV vaccine. Our insect cell system expressing chIL-12 effectively stimulated chicken splenocytes to secret IFN-γ (Figure 8). Therefore, the *Sf*9 cell-expressed rchIL-12 described here is easy to purify and utilize with other poultry vaccines. Reports have shown that recombinant IL-12 or IL-12 plasmids enhance the efficacy of human or animal vaccine candidates [34,35,36]. Therefore, IL-12 is a potential adjuvant for human or animal vaccines. Concentrations of chIL-12 above 2 ng did not stimulate a higher IFN-γ in chicken splenocytes, which is consistent with previous studies describing a negative regulatory response in chickens treated with high concentrations of chIL-12 [27,37]. 

Animal VLP vaccines are efficient in stimulating both cellular and humoral immune responses [38]. Indeed, the splenocytes collected from the VLP-inoculated chickens secreted IFN-γ and induced cell proliferation after treatment with purified rVP1, revealing that cell-mediated immune responses were elicited in the vaccinated chickens, particularly in the chIL-12 co-administrated groups. These observations suggest that, in combination with rchIL-12, the recombinant CAV vaccine induced systemic immunity in vaccinated chickens.

In conclusion, this report describes a baculovirus expression system generating CAV VLPs. Moreover, the chIL-12 expressed in this system served as an adjuvant to improve immune responses in vaccinated chickens, and this subunit vaccine induced much higher antibody titers than a commercial vaccine. In sum, this vaccine candidate may provide a more effective vaccination without the drawbacks of live attenuated CAV vaccines. In the future, this CAV vaccine will be optimized for large-scale production and use in the field.

## 4. Materials and Methods

### 4.1. Gene Cloning and Virus

CAV strain 1207PT05 (accession no: KX772161), isolated from a layer farm in our laboratory, was propagated in MDCC-MSB1 cells, and sequenced in our previous study [39]. ChIL-12-encoding regions containing p35 and p40 subunits cloned in the pET 32a vector were kindly provided by Dr. Long-Huw Lee (College of Veterinary Medicine, National Chung Hsing University), as previously described [27]. *VP1*, *VP2*, and *chIL-12* codon-optimized genes were synthesized and cloned into pUC57 plasmids (AllBio Co., Taichung, Taiwan). Sequences of 6 x His-tag were also inserted at the 3’-ends of *VP1* and *chIL-12*.

### 4.2. Animals

SPF chickens used for producing CAV anti-serum or animal experiments were obtained from the Animal Health Research Institute, Council of Agriculture, Taiwan. All of the SPF chickens were reared in positive pressure, air-filtered isolators. Feed and water were provided ad libitum. All of the animal experiments were approved by the Institutional Animal Care and Use Committee, National Chung Hsing University (IACUC no. 107-090), and performed on the basis of the Ethical Rules and Laws of the University.

### 4.3. Construction of Recombinant Baculoviruses

Codon-optimized target genes were amplified by PCR using primers with appropriate restriction enzymes (Table 1), and the PCR products were inserted into the pFastBac^TM^ Dual vector. In brief, the pFB-chIL-12 plasmid contained *chIL-12* under the control of the polyhedron (PH) promoter, and the *EGFP* gene, amplified from pcDNA™3.1 vector (Invitrogen, USA), was cloned downstream of the p10 promoter as a selection marker. The pFB-VP1/VP2 plasmid, which contained CAV *VP1* and *VP2* genes at independently separated expression cassettes driven by the PH promoters and the *EGFP* gene, was inserted in the vector, as described above (Figure 1). The recombinant plasmids were verified by sequencing, and transformed into MAX Efficiency^®^ DH10BAC^TM^ competent cells (Thermo, USA) to produce bacmids. The recombinant baculoviruses, B-chIL-12 and B-VP1/-VP2, were generated using the Bac-to-Bac^TM^ Baculovirus Expression System (Invitrogen, USA), following the manufacturer’s directions. *Sf*9 cells were used to generate recombinant baculoviruses and protein production via culture in a Sf-900^TM^ II media (Invitrogen, USA) at 27 °C.

### 4.4. Western Blot Analysis

The recombinant baculovirus-infected *Sf*9 cells were collected 48 h post infection (hpi). The cell lysates were re-suspended in equal volumes of a 2× Laemmli sample buffer (Bio-Rad, USA), separated by 12% SDS-PAGE, and transferred onto nitrocellulose membranes using a semi-dry transfer cell (Bio-Rad, USA). The membranes were then blocked with TBST (10 mM Tris-HCl, 150 mM NaCl, 0.5% Tween 20) containing 5% skim-milk powder. The recombinant VP1 (rVP1) was detected by Western blotting, using an anti-6X His tag^®^ monoclonal antibody (Abcam, USA) or chicken anti-CAV polyclonal antibodies as the primary antibodies. The expression of rVP2 was also verified by polyclonal antibodies recognizing *E. coli*-expressed VP2 as the primary antibody. In addition, the detection of recombinant chIL-12 by Western blotting was performed with anti-6X His tag^®^ monoclonal antibody (Abcam, USA) or polyclonal antibodies recognizing *E. coli*-expressed chIL-12 as the primary antibodies [27]. Alkaline phosphatase-conjugated goat-anti-mouse or -chicken IgG (KPL, USA) were used as the secondary antibodies to label the protein bands.

### 4.5. Indirect Immunofluorescence Assay (IFA)

*Sf*9 cells were cultured in six-well plates (Nunc, USA) at a density of 1.5 × 10^6^/mL, and infected with one of the recombinant baculoviruses, B-VP1/B-VP2 or B-chIL-12, at a multiplicity of infection (MOI) of 1. At 3 dpi, the infected cells were fixed with a 1:1 mixture of acetone and methanol for 30 min at 4 °C. After washing with phosphate-buffered saline (PBS) three times, the fixed cells were treated with chicken anti-CAV polyclonal antibodies, anti-6X His tag^®^ monoclonal antibody (Abcam, USA), or antibodies recognizing *E. coli*-expressed chIL-12 as the primary antibodies. Alex Fluor 594-conjugated goat anti-chicken IgG H&L (Abcam, USA) or Alex Fluor 594-conjugated goat anti-mouse IgG H&L (Abcam, USA) was used as the secondary antibody for the B-VP1/B-VP2 infected *Sf*9 cells to detect rVP1, respectively. The cells infected by B-chIL-12 were detected with the Alex Fluor 555-conjugated goat anti-mouse IgG H&L (Abcam, USA) as the secondary antibody. The cells were visualized using a fluorescence microscope (DM IRB, Leica, Germany).

### 4.6. Purification of Recombinant VP1 and chIL-12

To purify the rVP1 or rchIL-12, *Sf*9 cells at a density of 10^6^/mL were suspension-cultured and infected with B-VP1/B-VP2 or B-chIL-12 at a MOI of 1. At 96 hpi, the cells were lysed using Insect PE LB^TM^ lysis buffer (G-Bioscience, USA), and were sonicated. The sample was then mixed with HisPur^TM^ Ni-NTA Resin (ThermoFisher, USA) and loaded into a column. The column was washed with a washing buffer (20 mM pH 7.9 Tris, 30 mM imidazole, 500 mM NaCl), and the bound protein was eluted with an elution buffer (25 mM Tris, 200 mM imidazole, 500 mM NaCl). The elute was collected and verified with Western blotting. The protein concentration was determined using a Protein Assay kit (Bio-Rad, USA). 

### 4.7. CAV VLP Observation by Electron Microscope

The B-VP1-/B-VP2-infected *Sf*9 cells were treated with a lysis buffer and were sonicated, then centrifuged at 15,000× *g* for 30 min at 4 °C. The supernatant was transferred to a centrifuge tube containing an equal volume of 40% sucrose, then centrifuged at 270,000× *g* for 8 h at 4 °C. The pellet containing purified VLP was re-suspended in 0.5 mL of Tris buffer and examined by TEM.

### 4.8. Sf9-Expressed chIL-12 Bioactivity Assay 

To verify the bioactivity of *Sf9*-expressed rchIL-12, the splenocytes from one-week-old SPF chickens were cultured in 96-well plates at a density of 5 × 10^5^ cells per well, and incubated in a RPMI-1640 medium (Gibco, USA) containing 10% fetal bovine serum (Hyclone, USA) and 1% antibiotic-antimycotic (Gibco, USA) at 37 °C with 5% CO_2_. Serial 10-fold diluted purified rchIL-12, insect cell lysate (mock), or 10 µg/mL of concanavalin A (Con A, Sigma, USA), an antigen-independent mitogen of T lymphocytes as a positive control, were added to the splenocytes in triplicate. After 72 h incubation, the supernatant was collected and the level of IFN-γ was detected using a Chicken Matched Antibody Pair kit (Invitrogen, USA), in accordance with the manufacturer’s instructions.

### 4.9. Immunization of Chickens

The rVP1 for immunization was generated by a suspension-culture of *Sf*9 cells at a density of 10^6^/mL in 100 mL of Sf-900^TM^ II medium (Invitrogen, USA), which were then infected with B-VP1/B-VP2 recombinant baculovirus at a MOI of 1. The VP1 vaccine comprised the cell lysate of B-VP1/VP2-infected *Sf*9 cells; 35 μg/dose of rVP1 was mixed with Montanide ISA-70-VG adjuvant (Seppic France) at a 3:7 *w*/*w* ratio. The VP1/IL-12(5) and VP1/IL-12(10) vaccines were prepared with the same method, namely: a 35 μg/dose of rVP1 combined with 5 ng/dose or 10 ng/dose of rchIL-12, respectively. All of the baculovirus-infected *Sf*9 cells used for the vaccine preparation were inactivated with binary ethyleneimine (BEI; sigma, USA) at a final concentration of 3 mM. 

Thirty six-week-old SPF chickens were randomly assigned to one of five groups. The chickens in groups one–three received one subcutaneous dose (0.5 mL) of VP1, VP1/IL-12(5), or VP1/IL-12(10) vaccines, respectively. The chickens in group four received one dose of Circomune^®^ (Ceva Animal Health, USA), according to the manufacturer’s directions. The chickens in group five received 0.5 mL of PBS as a negative control. Two weeks after the primary vaccination, the chickens in each group were boosted with the same dose of vaccines. The blood samples were collected from a wing vein every seven days, and assessed using a ProFLOK AB Chicken Anemia Virus Antibody Test Kit (Zoetis, USA). At 28 days post-primary vaccination, the chickens were humanely slaughtered and their spleens were collected aseptically for splenocyte assays (below).

### 4.10. Splenocyte Proliferation Assay

The splenocytes collected from vaccinated chickens were seeded in 96-well plates at a density of 5 × 10^5^ cells/well. The cells were treated in triplicate with 1 μg/well of purified rVP1, 10 μg/mL of ConA (Sigma, USA), or PBS, and incubated at 37 °C with 5% CO_2_ for 72 h. Twenty microliter of 3-(4,5-Dimethylthiazol-2-yl)-2,5-diphenyltetrazolium bromide (MTT) at a final concentration of 5 mg/mL was added to the wells, and, and after 4 h, 100 µL of dimethyl sulfoxide (DMSO, Thermo, USA) was added to stop the reaction. Cell proliferation was detected with a Multiskan^TM^ FC Microplate Photometer (Thermo Scientific, USA). The optical density of each well was read at 450 nm. The proliferation response was represented by the SI—mean of experimental wells/mean of control wells. The supernatant of these treated splenocytes was also collected for IFN-γ quantification, as described above. 

### 4.11. Statistical Analysis

The data were analyzed with one-way analysis of variance (ANOVA) using Tukey’s test for the relative difference between the means of each group. All of the differences were considered significant at *p* < 0.05.

## Figures and Tables

**Table 1:** Oligonucleotide primers used in this study.

Target Gene	Primer	Sequence (5’→3’)	Amplicon Size (bp)	Restrict Enzyme
**CAV-VP1 ^1^**	BVP1-EcoRI-F	GAATTCATGGCTCGTCGCGCTC	1380	*Eco*RI
	BVP1-SpeI-R	ACTAGTTCAGTGGTGATGATGGTGATGA		*Spe*I
**CAV-VP2 ^1^**	BVP2-BamHI-F	GGATCCATGCACGGTAACGGTGG	665	*Bam*HI
	BVP2-NotI-R	GCGGCCGCTCACACGATG		*Not*I
**Chicken IL-12 ^1^**	BChIL-12-XhoI-F	CTCGAGATGTCCCACCTGCTGTT	1488	*Xho*I
	BChIL-12-KpnI-R	GGTACCTTAGTGATGATGGTGGTGGTG		*Kpn*I
**EGFP**	EGFP-SmaI-F	CCCGGGATGGTGAGCAAGGGCGAGGA	720	*Sma*I
	EGFP-SphI-R	GCATGCTTACTTGTACAGCTCGTCCATGCCGA		*Sph*I

^1^ The primer sequences of CAV-VP1, CAV-VP2, and chicken IL-12 are designed according to the optimized codons. Sequences of restriction enzymes are present underlined.
